# Raw meat based diet influences faecal microbiome and end products of fermentation in healthy dogs

**DOI:** 10.1186/s12917-017-0981-z

**Published:** 2017-02-28

**Authors:** Misa Sandri, Simeone Dal Monego, Giuseppe Conte, Sandy Sgorlon, Bruno Stefanon

**Affiliations:** 1 Department of AgroFood, Environmental and Animal Sciences, University of Udine, Via delle Scienze 2908, 33100 Udine, Italy; 2 Cluster in Biomedicine, CBM S.c.r.l., Bioinformatic Services, Area Science Park, I‑34149 Basovizza, Italy; 3 Department of Agricultural, Food and Agro-Environmental Sciences, University of Pisa, Via del Borghetto 80, 56124 Pisa, Italy

**Keywords:** Dog, Diet, Raw meat, Feces, Microbiome, Short chain fatty acids, Lactic acid

## Abstract

**Background:**

Dietary intervention studies are required to deeper understand the variability of gut microbial ecosystem in healthy dogs under different feeding conditions and to improve diet formulations. The aim of the study was to investigate in dogs the influence of a raw based diet supplemented with vegetable foods on faecal microbiome in comparison with extruded food.

**Methods:**

Eight healthy adult Boxer dogs were recruited and randomly divided in two experimental blocks of 4 individuals. Dogs were regularly fed a commercial extruded diet (RD) and starting from the beginning of the trial, one group received the raw based diet (MD) and the other group continued to be fed with the RD diet (CD) for a fortnight. After 14 days, the two groups were inverted, the CD group shifted to the MD and the MD shifted to the CD, for the next 14 days. Faeces were collected at the beginning of the study (T0), after 14 days (T14) before the change of diet and at the end of experimental period (T28) for DNA extraction and analysis of metagenome by sequencing 16SrRNA V3 and V4 regions, short chain fatty acids (SCFA), lactate and faecal score.

**Results:**

A decreased proportion of *Lactobacillus*, *Paralactobacillus* (*P* < 0.01) and *Prevotella* (*P* < 0.05) genera was observed in the MD group while Shannon biodiversity Index significantly increased (3.31 ± 0.15) in comparison to the RD group (2.92 ± 0.31; *P* < 0.05). The MD diet significantly (*P* < 0.05) decreased the Faecal Score and increased the lactic acid concentration in the feces in comparison to the RD treatment (*P* < 0.01). Faecal acetate was negatively correlated with *Escherichia/Shigella* and *Megamonas* (*P* < 0.01), whilst butyrate was positively correlated with *Blautia* and *Peptococcus* (*P* < 0.05). Positive correlations were found between lactate and *Megamonas* (*P* < 0.05), *Escherichia/Shigella* (*P* < 0.01) and *Lactococcus* (*P* < 0.01).

**Conclusion:**

These results suggest that the diet composition modifies faecal microbial composition and end products of fermentation. The administration of MD diet promoted a more balanced growth of bacterial communities and a positive change in the readouts of healthy gut functions in comparison to RD diet.

**Electronic supplementary material:**

The online version of this article (doi:10.1186/s12917-017-0981-z) contains supplementary material, which is available to authorized users.

## Background

Faecal microbiome in humans as well in animals is affected by several factors [1–3] and, among the others, diet and clinical conditions are likely the most important in dogs [4].

Clinical studies on dogs highlighted that the most recurrent faecal microbiome changes associated to gastro intestinal pathological conditions are typically a drop of biodiversity, an under or overgrowth of some distinct microbial communities and poor faecal quality [5–7]. However, an unequivocal identification of bad and good microbes at the different taxonomic level is not reported yet, since clinical-observational studies can intrinsically be biased from the difficulty to control some of the several confounding factors affecting gut microbiome of healthy and unhealthy dogs, as diet compositions, breed, gender, age, environmental and living conditions.

Recently, research has been carried out to clarify the role of diet on the modulation of faecal microbiome [8–13]. The studies have also highlighted the role of the intestinal microbiota in energy harvesting and in obesity development in dogs [14, 15] as in humans [16]. However, in these studies a large inter individual variability has been observed, suggesting that several other factors can influence the intestinal microbiome of dogs, which require to be understood and considered in population studies.

Dietary intervention studies are thus required to investigate the composition and the fluctuations of microbial community in healthy animals, to better understand the variability of gut microbial ecosystem under different feeding conditions, to improve diet design, to identify disease biomarkers and to develop target drug therapy [4].

Considering that several factors can affect gut microbiota, we sought to examine the effect of an abrupt change from extruded to raw meat based diet on the fluctuation of faecal microbial community, end product of fermentations and stool quality in a case control study in adult Boxer dogs. The approach used in the study is aimed at testing whether the change of dietary ingredients can modify faecal microbiome and whether the return to the initial dietary regime can re-establish the microbial profile.

## Methods

### Animals and housing

Eight healthy adult Boxer dogs housed in the same kennel, 5 females and 3 males, aged 4.2 ± 2.8 years, were recruited for the study. There was a couple of half sib dogs, male and female, which were allocated to each experimental group, whilst the others subjects were unrelated. Dogs were housed in pairs in 6x3 m enclosures, where a 2×3 m roof covered the paved portion of the pen. The sheltered areas were provided with beds for each dog and were used also for feeding, with water always available. The study was conducted in late autumn in North-East Italy, with an average temperature during the period of 10–15 °C and 60–70% relative humidity. During the day the dogs in pairs were allowed to exercise in 10×20 m green areas. At the beginning of the study, the average live weight was 30.3 ± 3 kg and all dogs had Body Condition Score (BCS) 4/9. The good clinical condition was confirmed by clinical examinations and blood biochemical analysis. All protocols, procedures and the care of the animals complied to the Italian legislation on animal care (DL n.116, 27/1/1992), and no ethical approval was required at the time the study was conducted.

### Diets

Up to the beginning of the study, the dogs had been fed a commercial extruded complete diet which was used as Reference diet (RD). The experimental diet (Mixed Diet, MD) was composed by raw human grade beef meat, representing about the 70% of the diet (w/w, for chemical composition see Table 1) added with a complement specifically formulated and manufactured for the study and provided by Nutrigene srl (Udine, Italy). A unique batch of raw meat was purchased for the trials, frozen at -20 °C and thawed every day. The complement was produced in one batch and was composed by rice flour, chickpeas flour, oat flakes, dry ground carrots, algae-derived Omega 3 fatty acids and mineral-vitamin complex. Chemical composition of the foods is showed in Table 1.

**Table 1:** Composition and nutritive value of diets and their constituents

Chemical composition		RD/CD	MD	Complement	Beef meat
Dry matter	%	90.0	57.6	93.0	35.5
Crude protein	%/DM	26.7	26.2	11.9	49.6
Crude fat	%/DM	10.6	18.2	4.1	41.4
Crude fiber	%/DM	2.8	0.7	1.2	-
Ash	%/DM	10.0	4.3	5.5	2.3
Ca	%/DM	0.90	0.70	1.16	0.04
P	%/DM	0.70	0.40	0.31	0.48
Metabolizable Energy	kcal/100 g DM	358	442	347	598

*RD* Reference Diet, extruded diet fed until the beginning of the experimental period (T0), *CD* The same RD diet used as Control Diet during the experiment, *MD* Experimental Mixed Diet

The MD was formulated to cover macro and micro nutritional requirements according to NRC recommendations [17]. Daily feed amounts and relative macronutrients supplied from the diets are reported in Table 2. Dogs were fed once daily at around 8:00 am. During the trial, the control group received the same amount of RD, which was also used as Control Diet (CD), while experimental diet was prepared by mixing the complement with the meat and adding water up to obtain a wet meal (approximatively, the ratio between water and complement was 2:1 w/w) and readily offered to the dogs.

**Table 2:** Daily dry matter and nutrients supplied by the diets

		RD/CD	MD^a^
Daily diets (g, as fed)		380	520
Nutrients			
Dry matter	g	342	300
Metabolizable Energy	kcal	1225	1269
Crude protein	g	91.2	78.5
Crude fat	g	36.1	54.6
Crude fiber	g	9.5	2.2
Ash	g	34.2	12.8
Carbohydrates (by difference)	g	171	151
Ca	g	3.4	2.2
P	g	3.1	1.1

^a^the daily mixed diet was composed by 200 g complement plus 320 g beef meat

*RD* Reference Diet, extruded diet fed until the beginning of the experimental period (T0), *CD* The same RD diet used as Control Diet during the experiment, *MD* Experimental Mixed Diet

### Experimental design

Dogs were randomly split in two groups of 4 individuals and allotted to experimental blocks. At the beginning of the trial (T0), one group received the MD and the other group continued to be fed with the CD for a fortnight (T14). After 14 days, the two groups were inverted, the Control group shifted to the MD and the other group shifted to the CD, for the following 14 days (T28). No transition period was applied to shift from the reference/control to the mixed diet. Individual live weight was also recorded at T14 and T28.

### Samples collection

Samples of faeces and blood were collected from each dog before the morning meal at the beginning of the study (T0), after 14 days (T14) before the change of diet and at the end of experimental period (T28). At each day of sampling, starting from 6:00 am the first stool evacuated from each dog was immediately and entirely collected with sterile gloves in hermetic sterile plastic bag. The plastic bags were immediately and entirely immersed in liquid nitrogen to frozen the stools until they arrived to the lab, then stored at -80 °C for the analysis. For the analysis, frozen stools were carefully cleaned from external contaminants with a sterile blade, then ground in a sterilized mortar under liquid nitrogen to avoid thawing and mixed. Two aliquots were obtained, placed in sterile plastic tube and stored at -80 °C for fatty acids and lactate or DNA analysis. From the cephalic vein, about 4 ml blood were collected for each sampling time, immediately divided into two aliquots, one with K3-EDTA and one without anticoagulant, stored at 8 °C until they arrived to the lab. Plasma and serum were separated by centrifugation for 25 min at 3250 rpm hence stored in 2.5 ml tubes at -20 °C until biochemical analysis.

### Blood analysis

Plasma and serum were sent under dry ice at the end of the trial to the certified laboratory of the Istituto Zooprofilattico delle Venezie (Legnaro, Padova, Italy) for biochemical analysis.

### Faecal DNA extraction, sequencing and taxonomic annotation

Prior to DNA extraction, faecal samples (150 mg) were washed following a 3-step washing procedure as described by Fortin et al. [18]. Microbial DNA of the faeces was extracted from 150 mg samples using a Faecal DNA MiniPrep kit (Zymo Research; Irvine, CA, USA) following the manufacturer’s instructions, including a bead beating step. Pre-amplification concentration of DNA in the samples was measured with a Nanodrop 3300 Spectrophotometer (Thermo Scientific; Waltham, MA, USA) and confirmed with a Qubit™ 3 Fluorometer (Thermo Scientific; Waltham, MA, USA) resulting in satisfactory quality and quantity. (219 ± 63 ng/μl, average 260/280 and 260/230 ratios 1.8 and 1.7, respectively). DNA was fragmented and 16SrRNA V3 and V4 regions amplified for library preparation, adding also the Indexes for sequencing, using a Nextera DNA Library Prep kit (Illumina; San Diego, CA, USA) following manufacturer’s instructions. 16S Amplicon PCR Forward Primer = 5' TCGTCGGCAG CGTCAGATGT GTATAAGAGA CAGCCTACGG GNGGCWGCAG 16S Amplicon PCR Reverse Primer = 5' and GTCTCGTGGG CTCGGAGATG TGTATAAGAG ACAGGACTAC HVGGGTATCT AATCC were used [19]. Around 460 bp amplicons were then sequenced with a MiSeq (Illumina; San Diego, CA, USA) in 2×300 paired-end mode following the standard procedures.

Sequenced reads that passed the quality check (Phred score ≥30) were then annotated for 16S rRNA taxonomic classification using the Ribosomal Database Project (RDP) Classifier, a Bayesian classifier developed to provide rapid taxonomic positioning based on rRNA sequence data [20]. The algorithm is a high-performance implementation of the RDP classifier described in Cole et al [21]. Data were lastly parsed and collected using a home prepared perl script (Additional file 1: Table S1).

### Faecal score, pH, lactate and fatty acids analysis

Right after evacuation, the stools were assigned a faecal quality score using a 5-points visual scale with 0.5 score interval ranging from 1 (hard and dry faeces) to 5 (liquid diarrhoea) [22]. Scores of 2–3 were considered the optimum, consisting in firm but not dry stool, with moderate segmentation visible, holding form when picked up leaving none or minimal residual on the ground.

After thawing, 2 g of faeces were mixed with 1/1 deionized water and pH measured using a Mettler Toledo InLab® Expert Pro pH meter. The analysis of short chain fatty acids (SCFA) (2:0, acetic; 3:0, propionic; 4:0, butyric; *iso* 4:0, isobutyric; 5:0, valeric; *iso* 5:0, isovaleric) and lactic acid of faecal samples was performed by HPLC according to the following procedures: 3 g of faeces was diluted with 150 mL of 0.1 *N* H_2_SO_4_ aqueous solution and homogenized for 2 min by UltraTurrax (IKA®-Werke GmbH & Co. KG, Staufen, Germany). The mix was centrifuged (5,000 × *g* for 15 min at 4 °C) to separate the liquid phase from the solid residuals and the liquid phase subsequently microfiltered (SLMV033RS, 0.45-μm Millex-HV, Merck-Millipore, Billerica, MA). The resulting sample was directly injected in the HPLC apparatus using an Aminex 85 HPX-87 H ion exclusion column (300 mm × 7.8 mm; 9-μm particle size; Bio-Rad, Milan, Italy) kept at 40 °C; the detection wavelength was 220 nm. The analyses were carried out applying an isocratic elution (flux 0.6 mL/min) with a 0.008 *N* H_2_SO_4_ solution as mobile phase; the injection loop was 20 μL. Individual SCFA and lactic acid were identified using a standard solution of 4.50 mg/mL of lactic acid, 5.40 mg/mL of acetic acid, 5.76 mg/mL of propionic acid, 7.02 mg/mL of butyric acid and isobutyric acid, 8.28 mg/mL of valeric acid and isovaleric acid in 0.1 *N* H2SO4 (69775, 338826, 402907, B103500, 58360, 75054, 129542, respectively; Sigma-Aldrich, Milano Italy). Quantification was done using an external calibration curve based on the standards described above.

### Statistical analysis

At each taxonomic level sequences for each sample were normalized to ‰ abundance profiles. Taxa with abundance lower than 10‰ [23] in more than 16 samples out of 24 were excluded from the statistical analysis. Shannon α-biodiversity (H’) index was also calculated at the genus level including all taxa according to the equation H’ = - sum(P_i *_ln P_i_), where P_i_ = frequency of every genus within the sample. Evenness index (J) was calculated as J = H’/ln S, where S = total number of genera within each sample.

The blood and faecal variables and metagenomics abundance were analyzed applying a Linear Mixed Model. The model included the fixed effect of time of sampling (3 levels, T0, T14 and T28), treatment (3 levels, RD, MD, CD), the interaction of time of sampling X treatment and the dog as random factor repeated over the time of sampling. Orthogonal contrasts of T14 *Vs* T0 and T28 *Vs* T0 were calculated and Least Significant Difference statistics with Bonferroni multiple testing correction on estimated marginal means were used as significance test. Pearson correlations between relative abundance of microbial families or genera and proportions of SCFAs and lactate were calculated. All statistical analysis were performed with SPSS Statistic [24].

## Results

### BCS and blood biochemistry

Dietary treatment did not affect significantly the body weight, which was equal to 30.1 ± 2.7 with CD and 29.9 ± 2.8 with MD, nor the BCS. For blood biochemistry (Additional file 2: Table S2), only plasma glucose was affected by MD (*P* < 0.05) and time of sampling (*P* < 0.05). The other parameters did not change significantly between groups.

### Metagenome sequencing and taxonomic annotation

An average of 337,224 ± 177,407 raw sequences were obtained for the samples. After the quality check, a mean of 362,292 ± 247,167, 297,745 ± 89,305 and 241,920 ± 50,365 sequences were available for taxonomic annotation for the RD, the MD and the CD groups, respectively. The bacterial annotations, the relative abundance across the dietetic treatments and the results of the statistical analysis are reported for the taxonomic levels of the Phylum, Family and Genus.

Dietary treatments had a significant effect on the phylum *Proteobacteria* (*P* < 0.05), which was higher in the MD compared to the RD (Table 3). An increased abundance was measured in the MD *Vs* RD also for the phyla *Actinobacteria* and *Fusobacteria* (*P* < 0.05). No difference were observed between CD and RD.

**Table 3:** Relative abundance (‰, annotated reads/1000 reads) of microbiome at a phylum taxonomic level in the faeces of dogs fed a Reference diet (RF), Mixed diet (MD) or Control diet (CD)

	RD		MD		CD		Effects		
	mean	st. dev.	mean	st. dev.	mean	st. dev.	treatment	MD vs RD	CD vs RD
Actinobacteria	10.06	2.67	39.57	37.92	9.25	7.08	Ns	*	Ns
Bacteroidetes	220.87	162.85	197.99	77.81	269.22	72.28	Ns	Ns	Ns
Firmicutes	705.42	190.43	608.12	133.72	618.64	82.48	Ns	Ns	Ns
Fusobacteria	46.69	22.16	109.55	50.90	77.29	8.21	Ns	**	Ns
Proteobacteria	13.02	10.00	43.63	12.66	23.85	8.47	*	**	Ns

*RD* Reference Diet, extruded diet fed until the beginning of the experimental period (T0), *CD* The same RD diet used as Control Diet during the experiment, *MD* Experimental Mixed Diet

Ns Not significant

*Significant for *P* < 0.05

**Significant for *P* < 0.01

At the family taxonomic level (Table 4), several bacterial families were significantly increased in the MD group. The effects of treatment and of the contrast MD *Vs* RD were significant for *Streptococcaceae*, *Clostridiaceae 1* and *Enterobacteriaceae*. For the *Bacteroidaceae*, *Veillonellaceae* and *Coriobacteriaceae*, significant effects were observed only for the MD *Vs* RD contrasts. A marked decrease (*P* < 0.01) of the *Lactobacillaceae* was observed as consequence of treatment and for MD *Vs* RD diets. Also the *Prevotellaceae* significantly changed across the diets (*P* < 0.05), being lower in MD and higher in CD, compared with the RD.

**Table 4:** Relative abundance (‰, annotated reads/1000 reads) of microbiome at a family taxonomic level in the faeces of dogs fed a Reference diet (RF), Mixed diet (MD) or Control diet (CD)

	RD		MD		CD		Effects		
	mean	st. dev.	mean	st. dev.	mean	st. dev.	treatment	MD vs RD	CD vs RD
Lactobacillaceae	313.44	143.27	9.56	12.45	219.04	109.11	**	**	Ns
Prevotellaceae	178.81	148.76	113.84	46.93	194.37	54.30	*	Ns	Ns
Peptostreptococcaceae	122.82	39.45	157.57	17.76	118.31	54.27	Ns	Ns	Ns
Lachnospiraceae	101.79	31.65	100.53	29.11	107.18	11.63	Ns	Ns	Ns
Fusobacteriaceae	46.67	22.15	109.51	50.88	77.27	8.21	Ns	**	Ns
Erysipelotrichaceae	54.29	24.34	76.51	32.28	43.63	26.11	Ns	Ns	Ns
Bacteroidaceae	27.75	16.96	63.30	52.96	60.60	21.33	Ns	*	Ns
Ruminococcaceae	31.68	11.99	22.74	7.74	45.10	11.60	**	Ns	*
Veillonellaceae	13.43	8.65	103.87	101.55	14.54	6.12	Ns	*	Ns
Acidaminococcaceae	10.11	12.07	15.74	6.13	13.04	3.76	Ns	Ns	Ns
Sutterellaceae	8.73	7.42	13.28	10.18	14.42	4.52	Ns	Ns	Ns
Streptococcaceae	11.97	8.52	53.58	56.37	10.40	8.97	*	**	Ns
Enterococcaceae	7.68	6.41	24.71	19.35	14.12	19.58	Ns	*	Ns
Peptococcaceae 1	9.99	5.15	13.78	6.87	10.37	3.39	Ns	Ns	Ns
Porphyromonadaceae	10.46	8.27	17.45	13.40	9.26	2.37	Ns	Ns	Ns
Coriobacteriaceae	7.72	3.25	16.16	8.20	6.95	3.07	Ns	*	Ns
Clostridiaceae 1	6.88	6.49	16.56	8.42	5.86	5.28	*	*	Ns
Enterobacteriaceae	0.47	0.26	24.54	8.85	1.98	0.82	**	**	Ns

*RD* Reference Diet, extruded diet fed until the beginning of the experimental period (T0), *CD* The same RD diet used as Control Diet during the experiment, *MD* Experimental Mixed Diet

Ns Not significant

*Significant for *P* < 0.05

**Significant for *P* < 0.01

**Fig. 1:**
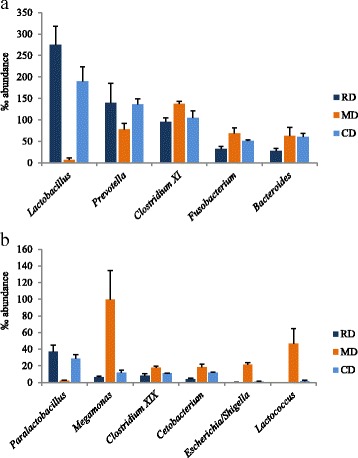
Abundance of faecal microbial genera (**a**), mean abundance higher than 50‰; (**b**), mean abundance lower than 50‰ significantly different in dogs fed MD, RD or CD diets. RD Reference Diet, extruded diet fed until the beginning of the experimental period (T0); CD The same RD diet used as Control Diet during the experiment; MD Experimental Mixed Diet. Data are reported as mean and standard deviation. *Clostridium XI, Bacteroides, Megamonas*: *P* < 0.05; *Fusobacterium, Clostridium XIX, Lactobacillus, Cetobacterium, Paralactobacillus, Escherichia/Sighella, Lactococcus*: *P* < 0.01

The abundance of the genera *Clostridium XI*, *Bacteroides* (*P* < 0.05), *Fusobacterium*, *Clostridium XIX*, *Cetobacterium*, *Escherichia/Sighella* and *Lactococcus* was significantly (*P* < 0.01) higher in MD diet compared to RD (Fig. 1; Additional file 3: Table S3). In the MD group, a marked decreased of the genera *Lactobacillus* and *Paralactobacillus* (*P* < 0.01) was observed. For the genus *Prevotella* a significant effect of the treatment was shown (*P* < 0.05), with a lower abundance in the MD group.

The effects of time and time X treatment were not significant at the Phylum (Table 3) or at the Family level (Table 4). At the Genus level, the relative abundance of *Clostridium XI* (*P* < 0.05) and *Turicibacter* (*P* < 0.01) significantly changed with time, and for *Sutterella* a significant effect was also observed for treatment (*P* < 0.01) and time X treatment interaction (*P* < 0.05) (Additional file 3: Table S3).

**Fig. 2:**
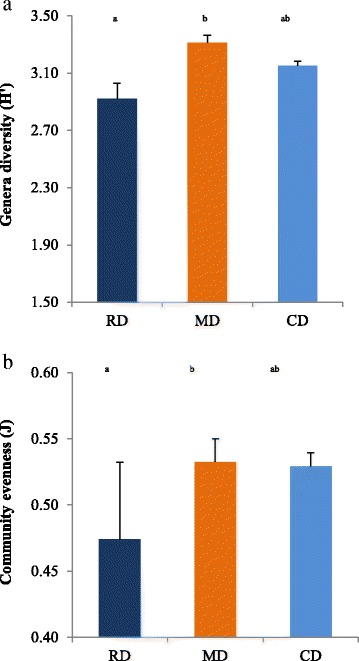
Indexes of H’ (**a**) and J (**b**) calculated from the abundances of genera for RF, MD or CD. RD Reference Diet, extruded diet fed until the beginning of the experimental period (T0); CD The same RD diet used as Control Diet during the experiment; MD Experimental Mixed Diet. Data are reported as mean and standard deviation. H’ Shannon alpha biodiversity index. J Evenness community index

The Shannon biodiversity Index (H’) at the genus level (Fig. 2a) showed a significant increase for the MD (3.31 ± 0.15) group in comparison to the RD group (2.92 ± 0.31; *P* < 0.05). It returned close to the RD in the CD treatment (3.15 ± 0.09). The same differences were observed also for the Evenness Index (J, Fig. 2b). In particular, the J value of the RD group was significantly lower than the MD and CD groups (*P* < 0.05).

### Faecal Score and end products of fermentation

**Fig. 3:**
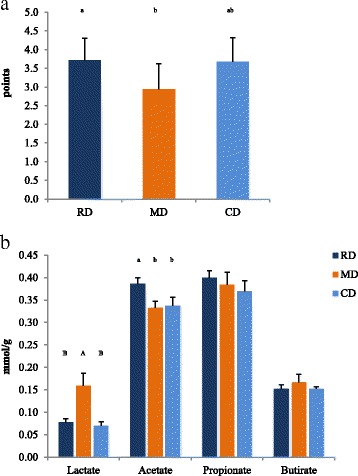
Faecal score (**a**), lactate and SCFA contents (**b**) in faeces of dogs fed RF, MD or CD. SCFA Short Chain Fatty Acids. RD Reference Diet, extruded diet fed until the beginning of the experimental period (T0); CD The same RD diet used as Control Diet during the experiment; MD Experimental Mixed Diet. Data are reported as mean and standard deviation. ^a, b^ *P* < 0.05; ^A,B^ *P* < 0.01

The MD treatment significantly (*P* < 0.05) lowered the Faecal Score and increased the lactic acid concentration in the feces in comparison to the RD treatment (*P* < 0.01) (Fig. 3a and b and Additional file 4: Table S4). A numerical increment, even though not significant (*P* = 0.081), was also observed for the proportion of butyrate in MD treatment. In comparison with the RD treatment, acetic acid was lower (*P* < 0.05) for MD and CD treatments, although for CD the concentration was closer to RD. No significant variations of molar content and proportion of the other SCFAs were observed.

### Correlations between metagenome, lactate and SCFAs proportions

Correlations analysis showed several significant effects between microbiome and SCFAs or lactate (Table 5). Acetate was negatively correlated with the genus *Escherichia/Shigella* (*P* < 0.01), belonging to the phylum *Proteobacteria*, with family *Lachnospiraceae* (*P* < 0.05) and the genus *Megamonas* (*P* < 0.01), belonging to the phylum *Firmicutes*. Positive correlations with butyrate production (*P* < 0.05) were calculated for the *Lachnospiraceae* and its genus *Blautia*, for the genus *Peptococcus* (phylum *Firmicutes*) and for the family *Coriobacteriaceae* (phylum *Actinobacteria*). Positive correlations with lactate production were observed for the genera *Megamonas* (*P* < 0.05) and *Escherichia/Shigella* (*P* < 0.01), for the family *Enterococcaceae* (*P* < 0.05) and the genus *Lactococcus* (*P* < 0.01) (phylum *Firmicutes*) and the genus *Clostridium XIX* (*P* < 0.05) (phylum *Fusobacteria)*. The genera *Lactobacillus* and *Paralactobacillus* in this study resulted negatively correlated with lactate (*P* < 0.01). For the SCFAs isoforms, positive correlations were calculated for isovalerate with the genus *Turicibacter* (*P* < 0.01) and for isobutyrate with the genera *Blautia* and *Sutterella* (*P* < 0.05).

**Table 5:** Significant correlation indexes between bacterial families or genera and lactate or SCFAs proportion

Family	Genus	Acetate, %	Isobutyrate, %	Butyrate, %	Isovalerate, %	Lactate, %
Coriobacteriaceae				0.496*		
Enterobacteriaceae	Escherichia/Shigella	-0.626**				0.823**
Enterococcaceae						0.528*
Erysipelotrichaceae	Turicibacter				0.573**	
Fusobacteriacee	Clostridium XIX					0.460*
Lachnospiraceae		-0.422*		0.510*		
Lachnospiraceae	Blautia		0.450*	0.460*		
Lactobacillaceae	Paralactobacillus					-0.558**
Lactobacillaceae	Lactobacillus					-0.568**
Peptococcaceae 1	Peptococcus			0.515*		
Streptococcaceae	Lactococcus					0.559**
Sutterellaceae	Sutterella		0.461*			
Veillonellaceae	Megamonas	-0.576**		0.504*		0.516*

*significant for *P* < 0.05

**significant for *P* < 0.01

Proportion is calculated as % of each acid on the sum of lactate and SCFAs

## Discussion

The influence of diet compositions on the modification of gut microbiome in dogs has been recently reviewed by Deng and Swanson [4]. Many of the reported studies concern changes in nutrients content, as proteins or fibers in dry extruded formulations, but only one study [25] investigated the composition of faecal microbiome in diets containing beef or chicken raw meats; however, also in this study a comparison with extruded kibbles was not carried out. The interest for raw meat-based diets has been increasing in the last years [26], since the nutritional properties of raw meats are thought higher than after extrusion [27]. According to Schlesinger and Joffe [28], the risks associated with feeding raw meat is controversial, and was reported only by in testimonials, case series or limited cohort and case-controlled studies. Our study is the first attempt to compare, in healthy dogs, a complete diet (MD), consisting of vegetable sources supplemented with vitamins and minerals and raw beef meat, with a commercial extruded diet (RD and CD). In our study, the diets were compared in terms of blood biochemistry, faecal quality, end products of fermentation and microbiome. To limit the variability of the meat source, in this study all dogs were offered only high grade skeletal muscle meat, originating from a single batch. The chemical composition reported in Table 1 was the average of 4 analysis. Published studies report adaptation periods varying from 10 days [11], 2 weeks [10, 25] to 4 weeks [9]. According to the results of these studies, and to avoid modifications due to unexpected environmental changes we applied a 14 d interval between the collection of samples.

The main phyla detected in the three diets (Table 3) corresponded to those reported for healthy dogs using other sequencing techniques [5, 6, 12, 29], but in our study a higher abundance of *Firmicutes* and lower abundance of *Bacteroidetes* were observed. Other studies report a large variability in the prevalence of these phyla, often with smaller abundance of *Firmicutes* and a greater prevalence of *Bacteroidetes* and *Fusobacteria* [14, 30]. Hence, a straight comparison of microbiome compositions with these and other published results appears difficult for the limited information available on diet compositions in these studies and for the different sequencing techniques used.

In the present study, MD diet significantly changed the abundance of the phyla *Actinobacteria*, *Fusobacteria* and *Proteobacteria*. However, at a phylum taxonomic level is difficult to understand the relationship between microbial communities and fermentation products and dietary regimes.

More evident was the effect of dietary shifts on the composition of microbial communities at the family taxonomic level. The inclusion of raw meat in the diet, together with the variation of composition and the physical form of MD, dramatically modified the abundance of the families *Lactobacillaceae*, *Fusobacteriaceae*, *Coriobacteriaceae*, *Clostridiaceae 1, Enterobacteriaceae, Streptococcaceae* and *Enterococcaceae* (Table 4).

Moderate variations of diet do not seem to influence intestinal microbial communities. The inclusion of navy beans in a control diet of healthy dogs did not caused a shift in faecal microbiome after 4 weeks of dietary intervention study [9]. Also Panasevich et al. [12] found limited variations in the composition of faecal microbiome increasing the potato fiber in the diet from 0 to 6%. A decreased proportion of the family *Coriobacteriaceae* was observed by Suchodolski et al. [5] in dogs with inflammatory bowel disease (IBD) and other faecal dysbiosis in comparison to healthy subjects, and Xenoulis et al. [31] observed a significant increase of *Enterobacteriaceae*, mainly due to *E. Coli* sequences in IBD affected dogs. However, these authors did not find changes in the families *Streptococcaceae, Enterococcaceae* and *Fusobacteriaceae*. The comparison of the present results with previously published data suggests that a relevant shift of faecal microbiota in healthy dogs can be observed only as a consequence of profound dietary variations.

The effect of the diets on microbial profile was more evident at the genus taxonomic level (Additional file 3: Table S3 and Fig. 1) and other significant variations for genera not included in the families significantly affected (Table 4) were found. Other than *Lactobacillus* and *Paralactobacillus* (family *Lactobacillaceae*), *Fusobacterium*, *Clostridium XIX* and *Cetobacterium* (family *Fusobacteriaceae*), *Escherichia*/*Shigella* (family *Enterobacteriaceae*), *Lactococcus* (family *Streptococcaceae*), diet significantly influenced the genera *Clostridium XI*, *Bacteroides* and *Megamonas*, but not their respective families. Of note, the relative abundance of these families and genera in the CD diet returned quite close to that of RD diet, further suggesting a dietary signature for microbiome as indicated also by Beloshapka et al. [25] and Hang et al. [32].

If the variations of microbiome observed in this study were associated or not to a better gut health is not easy to assess, but the increase of H’ in the MD diet, due to a better distribution of evenness J (Fig. 2a and b), would indicate an enhancement of gut health. Lower H’ and J in IBD affected dogs are reported by Suchodolski et al. [5, 33]. According to Alcock et al. [34], lower biodiversity of intestinal microbiome is associated to a higher microbial fitness, which is detrimental for host fitness, leading in mice and humans to unhealthy eating behavior and obesity. The relationship between biodiversity and obesity was also observed in Beagle dogs by Park et al. [15].

In favor of a better gut health for the raw meat-based diet (MD), was the improvement of faecal score (Fig. 3a), which further indicated a better colonic health, as suggested by Gagnè et al. [35]. Moreover, from the visual appraisal of the faecal output, which was observed to be reduced in the MD diet, a better apparent digestibility of the diet can be supposed, as also suggested by Beloshapka et al. [27] for dogs fed with raw meat.

As a further evaluation of microbiome community in the gut, we measured faecal SCFAs and lactate, since their concentration depends upon the colonic fermentation of the nutrients by microorganisms [36, 37].

Dogs can digest starch in the small intestine [38] and bacteria can ferment undigested starch and others complex carbohydrates in the large intestine producing SCFAs. Even though the contribution of these end products of fermentation for the energy balance of the host is considered marginal in dogs [37], the SCFAs are important growth factors for intestinal cells and for gut health [39], having also immunoregulatory T cells activity [40].

The average content of faecal SCFAs ranged from 195.7 to 216.9 μmol/g, a level generally found in animal fed low fiber diets [27, 41]. Amount, type and physical form of the fiber substrates affect the extent and the end-products of the fermentation [12]. However, in our trial total SCFAs were not affected by diet (Additional file 4: Table S4) even though the amount of crude fiber supplied with RD and CD the diets was higher than that provided by MD diet (Table 2). This can be the combined result of a reduced fermentation of the fiber after extrusion together with an increase of the intestinal transit time of RD and CD diet due to the higher crude fiber content.

Overall, SCFAs profile measured in the present research resulted similar to that reported for healthy dogs in a previous study [41]. Correlations analysis between the abundance with specific families and genera with SCFAs and lactate proportion in the faeces (Table 5) confirmed a statistical, although not biochemically proven, association of some microbial taxa to the end products of fermentation. However, caution must be taken before assessing a direct link between one microbial taxa and end products of fermentation. Gut microbial ecosystem is complex, presenting a mixture of common and divergent interests, with competition or mutual benefits, in a way that some product of fermentation from one microbial strain can be the substrate for another strain, sometimes occupying the same ecological niche [34].

There was a positive correlation between members of the family *Coriobacteriaceae* and with the family *Lachnospiraceae* (notably the genera *Blautia* and *Peptococcus*) with butyrate, supporting a positive role of these microbes on gut health. Butyrate is an essential substrate for cells of intestinal mucosa [37, 42] and the increase of its content in gut can influence other physiological effect at a whole organism level [42, 43].

Another very interesting correlation was calculated for the genus *Megamonas*, since other than increasing faecal butyrate also caused a shift between acetate and lactate, with a positive correlation with this latter acid. *Megamonas*, a predominant genus of the family *Veillonellacee*, is reported to increase in the faeces of dogs fed with diet supplemented with inulin [25] or fructooligosaccharides [44], suggesting a potential impact of this bacteria on gastrointestinal health.

The specific role of acetate remains poorly known and still under investigation in mammals. Acetate in dogs is produced by the fermentation of fiber [11] or from undigested protein in the colon [45]. In humans and in mice the increase of acetate produced from *Bifidobacterium* has been reported to protect the host from enteropathogenic infection via carbohydrate transporters [46]. In the present study we did not observed a significant variation of acetate concentration between CD and MD, neither a changed abundance of *Bifidobacteria* consequent to the experimental diet.

Acetate has also been reported to stimulate insulin secretion and related changes associated with obesity and metabolic syndrome [47]. In mice, Frost et al., [48] observed a reduction of appetite through the interaction with the central nervous system after peripheral administration of acetate, without differences in plasma glucose, peptide YY (the anorexogenic gut hormone PYY) and GLP-1 (glucagon-like peptide-1). In dogs, Bosch et al. [49] reported a reduction of voluntary intake associated to higher acetate in faeces, but they did not observe any effect in the postprandial plasma glucose, PYY, GLP-1 and ghrelin responses.

These conflicting evidences deserve further studies to clarify the physiological role of acetate, especially in dogs. The importance to consider the microbial community as a whole is evident from the concurrent effect on lactate proportion of *Escherichia/Shigella* (*P* < 0.01), *Enterococcaceae* (*P* < 0.05), *Clostridium XIX* (*P* < 0.05) and, especially, of omeolactic bacteria *Paralactobacillus*, *Lactobacillus* and *Lactococcus.* Microbes of the family *Lactobacillacae* are generally associated with higher lactate, but in our dietary intervention study *Lactobacillus* almost disappeared in the raw met diet (MD). Instead, *Lactococcus*, another lactic acid genus poorly observed in other studies [10, 12, 29], strongly increased in the MD diet, probably occupying the ecological niche that in the extruded foods (RD and CD diets) are usually a more suitable environment for *Lactobacillacae.* MD diet supplied less, but higher digestible starch compared with the RD diet (Table 2, carbohydrates by difference), and in the complement the starch from rice and chickpeas was thermal treated and highly gelatinized, being probably more accessible for fermentations.

Since Bazolli et al. [36] reported that an increase of lactate in faeces can be related to carbohydrates escaping duodenal digestion, the observed increase of lactate in MD diet was probably the results of the variation of microbial community. It has been shown that excessive concentration of lactate leads to a higher osmotic pressure in the intestinal lumen with consequent increase of faecal volume, moisture content and subsequent poor faecal quality [50, 51]. In our study, only the molar proportion of lactate changed (Fig. 3), without a significant difference in the total amount of SCFAs and faecal pH. The concomitant reduction of the Faecal Score would indicate that the increase of lactate was related with a better gut health, as reported by Swanson et al., [37]. Furthermore, Felix et al. [52] observed that faecal lactate is related with lactic acid-producing microorganisms, which can inhibit the development of proteolytic bacteria, in the gut of the dogs.

## Conclusions

The studies on the composition and variation of faecal microbiome in healthy dogs offer a promising opportunity to better understand the factors affecting the microbial communities and the end products of fermentations, but further efforts from the scientific community are required to clarify if a reference compositions for healthy dogs can be assessed.

From our results and from the comparison with existing scientific evidences, it appears that the modification of microbiome can be attained when a considerable variation of dietary regimes is applied. Specifically, the administration of highly digestible feed, combining fresh meat with readily fermentable substrates, promoted a more balanced growth of bacterial communities and a positive change in some of the readouts of healthy gut functions.

## Additional files

Additional file 1: Table S1. Script used for parsing and collecting metagenomic data. (XLS 28 kb)
Additional file 2: Table S2. Blood biochemistry of dogs fed a Reference diet (RF), Mixed diet (MD) or Control diet (CD). Means, standard deviations and statistical effects are reported for the three diets. (XLSX 12 kb)
Additional file 3: Table S3. Relative abundance (‰, annotated reads/1000 reads) of microbiome at a genus taxonomic level in the feces of dogs fed a Reference diet (RF), Mixed diet (MD) or Control diet (CD). Means, standard deviations and statistical effects are reported for the three diets. (XLSX 13 kb)
Additional file 4: Table S4. Fecal score and pH, lactate and SCFAs of dogs fed a Reference diet (RF), Mixed diet (MD) or Control diet (CD). Means, standard deviations and statistical effects are reported for the three diets. (XLSX 12 kb)

## Abbreviations

CD: The same RD diet used as control diet during the experiment
H’: Shannon α-biodiversity index
IBD: Inflammatory bowel disease
J: Evenness index
MD: Experimental mixed diet
RD: Reference diet, extruded diet fed until the beginning of the experimental period
RDP: Ribosomal database project
SCFA: Short chain fatty acids
T0: Time of sampling at day 0, beginning of study
T14: Time of sampling at day 14, change of groups
T28: Time of sampling at day 28, end of study
